# Efficacy of Veno-Arterial Extracorporeal Life Support in Adult Patients with Refractory Cardiogenic Shock

**DOI:** 10.1177/11795484221113988

**Published:** 2022-07-21

**Authors:** ER Kurniawati, SMJ van Kuijk, NPA Vranken, JG Maessen, PW Weerwind

**Affiliations:** 1 Department of Cardiothoracic Surgery, 199236 Maastricht University Medical Center+, Maastricht, The Netherlands; 2 Department of Clinical Epidemiology and Medical Technology Assessment, 199236 Maastricht University Medical Center+, Maastricht, The Netherlands; 3 Department of Cardiology, 3802 Zuyderland Medical Center, Heerlen, The Netherlands; 4 Cardiovascular Research Institute Maastricht (CARIM), 5211 Maastricht University, Maastricht, The Netherlands

**Keywords:** refractory cardiogenic shock, extracorporeal life support, oxygen delivery, lactate, lactate clearance, pH, 30-day survival

## Abstract

**Background:**

This study aimed to describe the efficacy of veno-arterial extracorporeal life support (VA-ECLS) through early lactate clearance and pH restoration and assess the potential association with 30-day survival following hospital discharge.

**Methods:**

Data of patients receiving VA-ECLS for at least 24 h were retrospectively compiled. Blood lactate levels, liver enzymes, and kidney parameters prior to VA-ECLS initiation and at 2, 8, 14, 20, and 26 h of support had been recorded as part of clinical care. The primary outcome was 30-day survival.

**Results:**

Of 77 patients who underwent VA-ECLS for refractory cardiogenic shock, 44.2% survived. For all non-survivors, ECLS was initiated after eight hours (*p* = .008). Blood pH was significantly higher in survivors compared to non-survivors at all time points except for pre-ECLS. Lactate levels were significantly lower in survivors (median range 1.95-4.70 vs 2.90-6.70 mmol/L for survivors vs non-survivors, respectively). Univariate and multivariate analyses indicated that blood pH at 24 h (OR 0.045, 95% CI: 0.005-0.448 for pH <7.35, *p* = .045) and lactate concentration pre-ECLS (OR 0.743, 95% CI: 0.590-0.936, *p* = .012) were reliable predictors for 30-day survival. Further, ischemic cardiogenic shock as ECLS indication showed 36.2% less lactate clearance compared to patients with other indications such as arrhythmia, postcardiotomy, and ECPR.

**Conclusion:**

ECLS showed to be an effective treatment in reducing blood lactate levels in patients suffering from refractory cardiogenic shock in which the outcome is influenced by the initial lactate level and pH in the early phase of the intervention.

## Background

During cardiogenic shock, oxygen delivery may reach far below tissue oxygen demands, resulting in the so-called oxygen debt phenomenon.^1^ Oxygen debt is defined as additional oxygen needed during the oxidative energy processes following tissue hypoxia to reconvert lactic acid to glucose, as well as decomposed adenosine triphosphate and creatine phosphate to their original states.^2^ Most organs and body tissues produce lactate even during aerobic circumstances. Therefore, the end product of glycolysis, ie, pyruvate, will mostly enter the Krebs cycle, and merely a small amount will contribute to lactate production. Approximately 60% of lactate is cleared by the liver and 25% to 30% by the kidneys.^3^ Metabolism shifts to anaerobic pathways and lactate production during anaerobic circumstances, resulting in hyperlactatemia and acidosis.^4^ As a marker of adequate tissue perfusion,^4^ lactate has proven to be a robust outcome predictor.^1^ In addition, hyperlactatemia and acidosis resulting from cardiogenic shock are aggravated by decreased liver and kidney function due to hypoperfusion.^4^

Oxygen debt repayment plays a key role in treating shock, which may be supported by timely initiation of veno-arterial extracorporeal life support (VA-ECLS) in cardiogenic shock patients.^5^ In these cases, VA-ECLS is an *ultima ratio* therapy for severe refractory cardiogenic shock. It enables stabilization of hemodynamics and circulation and allows treatment of the underlying disease while the cardiac function is recovering.^6^ Rapid lactate clearance during VA-ECLS may, therefore, indicate tissue perfusion recovery and oxygen debt repayment.^7^ Thus, serial measurements of factors reflecting metabolic acidosis (lactate and pH) during VA-ECLS may allow evaluation of the effectiveness of circulatory support and, consequently, clinical outcome.

This study aimed to describe the efficacy of VA-ECLS through lactate clearance and pH restoration. Additionally, hepatic and renal function biomarkers were taken into account to assess the potential association of lactate clearance with 30-day survival following hospital discharge.

## Methods

Retrospective data acquisition and analyses were performed anonymously in accordance with the Dutch law for approving medical research and included adult patients receiving VA-ECLS between August 2012 and January 2018. The indication for VA-ECLS was a severe refractory cardiogenic shock, ie, poor cardiac contractility despite percutaneous coronary intervention, adequate filling volume, administration of inotropes, and use of an intra-aortic balloon pump or failure to wean from cardiopulmonary bypass. In our medical center, a multispecialty team consisting of an intensivist, a cardiologist, a cardiothoracic surgeon, and a perfusionist decided to initiate ECLS. Only patients who underwent VA-ECLS therapy for at least 24 h were included in this study. Patients with previously known irreversible brain damage, uncontrolled bleeding or contraindication to anticoagulation, terminal malignancy, and refractory cardiac arrest before initiation of VA-ECLS were excluded. Another exclusion criterion was baseline lactate values of less than 2 mmol/L. The medical ethics committee of the University Hospital Maastricht and Maastricht University (METC) approved this study (project 300751, entitled “the efficacy of extracorporeal life support”) as applicable to non-medical research involving human subjects act (non-WMO). Therefore, the necessity of informed consent was waived.

### Procedure and management of VA-ECLS

The ECLS procedure and management have been described previously.^6^ In short, the extracorporeal system consisted of vascular access (cannulae), heparin-coated tubing circuitry, a centrifugal blood pump, and an oxygenator (Bioline-coated PLS system or Cardiohelp system, Maquet Cardiopulmonary AG, Getinge Group, Rastatt, Germany). All cannulation were performed via femoral access by either surgical cutdown or percutaneous puncture. Postcardiotomy patients underwent over cannulation to the femoral vessels as the sternum was always closed to minimize bleeding complications. The tip of the arterial cannula (19 or 21 Fr Carmeda bio-active-coated Bio-Medicus femoral cannula, Medtronic Inc., Minneapolis, USA) was placed at the aortoiliac junction. In contrast, the tip of the venous cannula (23 or 25 Fr Carmeda-coated Bio-Medicus femoral cannula, Medtronic Inc.) was set in the right atrium. In addition to accessing the femoral artery, an 8 or 10 Fr Carmeda-coated pediatric cannula (Medtronic Inc.) was placed as a side branch from the arterial cannula to facilitate antegrade, distal limb perfusion, as described elsewhere.^8^ A continuous heparin infusion provided the necessary systemic anticoagulation and was adjusted accordingly to keep the activated partial thromboplastin time (aPTT) between 50 and 80 s. In the case of bleeding, the heparin infusion was stopped, allowing the aPTT to drop to a near-normal level (45-55 s).

Extracorporeal blood flow was adjusted to maintain adequate systemic blood flow and oxygen supply as monitored by the mean arterial pressure and lactate concentrations. To preserve organ function, fluids were infused to maintain a mean blood pressure of >70 mm Hg and a blood flow rate of 3.5 to 5.0 L/min. Inotrope dosages were adjusted over time based on the desired mean arterial pressure. A transthoracic and/or transesophageal echocardiogram was performed daily during ECLS to estimate myocardial recovery and detect possible ventricular cavity thrombus formation.

Weaning was defined as the successful separation from ECLS requiring either minimal inotropic support or ventilation support. The weaning procedure applied in our medical center has been described previously.^9^ No weaning attempts were undertaken in the first 24 h after ECLS initiation. Mortality was defined as death from any cause within 30 days following VA-ECLS initiation.

### Data acquisition

All clinical and laboratory variables of patients requiring VA-ECLS for severe refractory cardiogenic shock were recorded in our institutional database. The parameters assessed in this study were oxygen delivery (DO_2_), blood lactate levels, aspartate aminotransferase (AST), alanine aminotransferase (ALT), lactate hydrogenase (LDH), creatine kinase (CK), creatinine, and estimated glomerular filtration rate (eGFR). These variables were analyzed at different timelines, including before ECLS initiation and at 2, 8, 14, 20, and 26 h after ECLS initiation.

Oxygen delivery (DO_2_) is defined as the oxygen carried by the blood to tissue and was calculated based on the following equation^10^:

$$DO_{2}=\mathrm{CO}\times (1.36\times Hb\times \mathrm{saturation})+(0.003\times PO_{2})$$

Where: CO = cardiac output (L/min), Hb = hemoglobin concentration (g/dL), PO_2_ = partial pressure of oxygen (mm Hg).

Total DO_2_ at a specific time point is the cumulative oxygen delivered from the beginning of the support until a certain time period.

The estimated glomerular filtration rate (eGFR) (mL/min/1.73 m^2^) was calculated according to the following equation^11^:

$$186\times (\mathrm{creatinine}(\mu \mathrm{mol}/L)/88.4{)}^{-1.154}\times (\mathrm{age}{)}^{-0.203}\times (0.742\;\mathrm{if}\;\mathrm{female})\times (1.210\;\mathrm{if}\;\mathrm{black})$$

The pH was measured before ECLS initiation, after two hours, and every six hours afterward. The indexed support flow (L/min/m^2^) was also taken into consideration.

Lactate clearance was defined as the lactate concentration before ECLS initiation (T_0_) minus the lactate concentration at a specific time point (T_x_) after ECLS initiation divided by the lactate concentration at T_0_ and multiplied by 100%. Thus, it can be calculated by the following calculation:

$$\mathrm{Lactate}\;\mathrm{clearance}\;\left(\mathrm{Tx}\right)=\frac{\mathrm{Lactate}\;\mathrm{value}\;\mathrm{before}\;\mathrm{ECLS}\;\mathrm{initiation}\;\left(T0\right)-\mathrm{Lactate}\;\mathrm{value}\;(\mathrm{Tx})}{\mathrm{Lactate}\;\mathrm{value}\;\mathrm{before}\;\mathrm{ECLS}\;\mathrm{initiation}\;\left(T0\right)}\times 100\text{\%}$$

A positive lactate clearance indicates a decrease in lactate concentration, while a negative lactate clearance indicates an increase.

### Outcomes

The primary outcome parameter was 30-day survival following hospital discharge and the secondary outcome concerned factors associated with lactate clearance at 24 h after ECLS initiation.

### Statistical analysis

All analyses were performed using SPSS (IBM SPSS Statistics Version 25). Results are presented as frequencies with percentages for categorical variables or as medians with interquartile ranges for continuous variables. The Kolmogorov-Smirnov test and visual evaluation using histograms were used to assess data distribution for all continuous parameters. Differences in categorical variables were analyzed by Chi-square or Fishers’ Exact test, when appropriate. Meanwhile, group differences in continuous variables for normally distributed parameters were analyzed using the independent-samples t-test. The paired-samples t-test was used to assess differences within the same patients across time points. Non-normally distributed continuous variables were compared using the non-parametric equivalent tests (*ie*, the Mann-Whitney U test and the Wilcoxon signed-rank test). Pearson's or Spearman's correlation coefficient was computed to estimate the univariable correlation between each variable and the outcomes. Multivariable or adjusted associations with 30-day survival were investigated with logistic regression analysis. Further, generalized estimating equations (GEE) were performed to examine the longitudinal effects of oxygen debt repayment on the lactate level of patients undergoing VA-ECLS. For all tests, a two-tailed *p*-value<.05 was considered statistically significant.

## Results

A total of 77 adult patients underwent VA-ECLS for refractory cardiogenic shock and consisted of predominantly male patients (65%). Demographic and ECLS characteristics are summarized in Table 1.

**Table 1.** Demographic and characteristics of patients requiring VA-ECLS for refractory cardiogenic shock

BASELINE	SURVIVORS (N = 34)	NON-SURVIVORS (N = 43)	TOTAL (N = 77)	*P*-VALUE
Age, years	62.0 [54.4-70.6]	66.5 [56.5-70.1]	65.3 [55.6-70.2]	.357
Gender (male, %)	21 (62%)	29 (67)	50 (65)	.604
BSA (m^2^)	1.95 [1.77-2.09]	1.96 [1.81-2.10]	1.96 [1.79-2.10]	.819
IABP (Yes/No)	11 (32.4%)/23 (67.6%)	12 (27.9%)/31 (72.1%)	23 (29.9%)/54 (70.1%)	.803
ECLS duration (hours)	128.6 [79.9-183.7]	115.0 [78.4-197.5]	116.8 [80.2-189.1]	.687
Decision to initiate ECLS within 8 h of onset of failure (Yes/No)	34 (100%)/ 0(0%)	35 (81.4%)/ 8 (18.6%)	69 (90%)/ 8 (10.4%)	.008
Mean support flow (L/min)	4.0 [3.7-4.4]	4.2 [3.8-4.4]	4.2 [3.7-4.4]	.432
ECLS indication (%): - Arrhythmia - Postcardiotomy - Ischemic cardiogenic shock - ECPR - Others (myocardiac stunning, tamponade)	23 (68%) 0 (0%) 1 (3%) 8 (24%) 2 (6%)	28 (65%) 3 (75) 0 (0%) 11 (26%) 1 (2%)	51 (66%) 3 (4%) 1 (1%) 19 (25%) 3 (4%)	.366

Median with interquartile range [IQR], BSA = Body Surface Area, Mean flow = flow from ECLS device to patient.

Thirty-four patients (44.2%) survived at 30 days after weaning from ECLS, while 55.8% died. The sample's median age was 65.3 years, with survivors younger than non-survivors (62.0 vs 66.5 years, respectively, *p* = .357). The study population's median BSA was 1.94 m^2^, with a minimum of 1.55 m^2^ and a maximum of 2.28 m^2^ (*p* = .819). The median duration of ECLS was 116.8 h, with a shorter median ECLS duration for non-survivors as compared to the survivors’ group (115.0 vs 128.6 h, *p* = .687). During the first 24 h of ECLS, the median support flow was slightly higher for non-survivors than survivors’ group (4.2 vs 4.0 L/min, *p* = .432). Arrhythmia was the main indication for ECLS in this cohort (66%). There were no significant differences in demographic characteristics between survivors and non-survivors except for the decision to initiate ECLS within 8 h, where 100% of those who did not meet this timeframe died within 30-day following hospital discharge (*p* = .008).

There was a significant difference in lactate and pH level before and at 24 h after ECLS initiation (*p* < .001), where lactate concentration before the initiation was higher than at 24 h after initiation, and *vice-versa* for blood pH. Lactate levels were significantly lower at all time points in survivors in comparison to non-survivors. Blood pH levels were significantly higher in survivors compared to non-survivors across all time points (*p* < .05) except for prior to ECLS initiation (*p* = .987). Concurrently, base excess (BE) was significantly lower in non-survivors than survivors at 8,14, 20, and 26 h. Urea, creatinine, and eGFR as kidney function markers were significantly different at various observation time between the two groups (urea at 2 and 26 h after ECLS initiation, creatinine and eGFR at 2 h after ECLS initiation). Furthermore, liver enzymes showed significant differences at various time-points (20 and 26 h for AST, 26 h for ALT, and 20 h for LDH) (Appendix 1).

Lactate clearance at all time points did not differ between survivors and non-survivors. Although non-significant, the median lactate clearance for survivors was higher than non-survivors throughout the ECLS duration and peaked at 62.20% and 50.63% for survivors and non-survivors, respectively (Table 2).

**Table 2.** Lactate clearance at all time points

	SURVIVORS	NON-SURVIVORS	*P*-VALUE
Lactate clearance 0–2 h	14.65% [−4.53%-28.30%]	7.31% [−4.65%-27.71%]	.466
Lactate clearance 0–8 h	40.59% [−9.94%-55.97%]	32.20% [−19.54%-52.53%]	.644
Lactate clearance 0–14 h	49.38% [−14.68%-65.46%]	40.58% [9.23%-57.06%]	.637
Lactate clearance 0–20 h	56.27% [15.63%-71.71%]	51.35% [−8.89%-72.31%]	.659
Lactate clearance 0–26 h	62.20% [35.35%-75.66%]	50.63% [17.24%-72.90%]	.330

The total DO_2_ was similar between survivors and non-survivors at all time points, with a slightly higher total DO_2_ for the survivors’ group except at 14 and 26 h after ECLS initiation (Table 3).

**Table 3.** Total DO_2_ at all time points

	SURVIVORS	NON-SURVIVORS	*P*-VALUE
DO_2_ 0–2 h	1049.85 [882.66-1247.79]	1017.05 [897.79-1250.77]	.644
DO_2_ 0–8 h	2961.69 [2367.85-3294.57]	2715.28 [2389.91-33082.67]	.395
DO_2_ 0–14 h	4574.93 [3802.11-5371.85]	4668.66 [3950.55-5189.45]	.682
DO_2_ 0–20 h	6387.10 [5792.76-7062.13]	6237.76 [5764.87-6669.76]	.330
DO_2_ 0–26 h	7576.62 [6979.11-7971.06]	7603.95 [7015.90-8700.59]	.573

Lactate clearance showed weak positive correlations with total DO_2_ at 2, 8, and 14 h but was not statistically significant (Table 4). Additionally, lactate clearance was negatively correlated with total DO_2_ at 26 h and showed a statistically significant difference at 26 h after ECLS initiation (r_s_(75) = -0.234, *p* = .040).

**Table 4.** Correlation between total lactate clearance and oxygen delivery at different time points

	RHO	*P*-VALUE
Lactate clearance and DO2 total at 2 h	0.170	.140
Lactate clearance and DO2 total at 8 h	0.186	.106
Lactate clearance and DO2 total at 14 h	0.155	.178
Lactate clearance and DO2 total at 20 h	−0.068	.559
Lactate clearance and DO2 total at 26 h	−0.234	.040

As part of multivariable logistic regression analysis, the independent predictors for 30-day survival were pH at 26 h (odds ratio [OR]: 0.045, 95% confidence interval [CI]: 0.005-0.448 for pH <7.35, *p* = .045) and lactate concentration before ECLS initiation (OR 0.743, 95% CI: 0.590-0.936, *p* = .012) (Table 5).

**Table 5.** Logistic regression analysis

VARIABLE	SURVIVORS (n = 34)	NON-SURVIVORS (n = 43)	UNIVARIATE ANALYSIS	MULTIVARIABLE ANALYSIS		
			*P*-VALUE	OR [95% CI]	RC	*P*-VALUE
pH at 26 h			.003			.024
pH <7.35 normal pH (7.35-7.45) pH >7.45	19 3 11	20 18 6		0.045 [0.005-0.448] ref 3.005 [0.352-25.637]	−3.091 ref 1.100	.008 ref .314
BE (mmol/L)	−1.37 ± 4.52	−4.17 ± 5.10	.014	1.008 [0.785-1.296]	0.008	.948
AST (U/L)	776.91 ± 1644.64	1715.1 ± 3566.0	.028	1.000 [1.000-1.001]	0.000	.232
Urea (mmol/L)	8.01 ± 2.86	11.44 ± 6.26	.022	0.988 [0.803-1.215]	−0.013	.906
Creatinine (μmol/L)	122.01 ± 54.53	168.49 ± 80.36	.007	0.997 [0.980-1.015]	−0.003	.767
eGFR (mL/mimn/1.73 m^2^)	61.05 ± 27.53	44.38 ± 22.64	.007	1.012 [0.969-1.058]	0.012	.581
Pre-lactate (mmol/L)	5.45 ± 3.46	7.24 ± 3.94	.018	0.743 [0.590-0.936]	−0.297	.012

ref: reference

Results of the multivariable linear regression analyses using GEE showed that ECLS indication was significantly associated with lactate clearance for VA-ECLS patients (Table 6). After adjustment, patients with ischemic cardiogenic shock as ECLS indication showed 36.2% less lactate clearance compared to patients with other indications such as arrhythmia, postcardiotomy, and ECPR (*p* < .001). Although the oxygen delivery was not statistically significantly associated with lactate clearance, for every 100 ml/min/m^2^ of oxygen delivered, lactate concentration decreased on average by 1.1% (*p* = .443).

**Table 6.** Longitudinal linear regression (GEE) models

VARIABLE	UNIVARIABLE		MULTIVARIABLE	
	RC (95% CI)	*P*-VALUE	RC (95% CI)	*P*-VALUE
ECLS indication		<.001		<.001
Arrhythmia Postcardiotomy Ischemic cardiogenic shock ECPR Others	1.9 [−26.4-30.2] 11.1 [−18.6-40.8] −14.5 [−42.6-13.6] 6.9 [−21.8-35.6] ref	.896 .464 .311 .636	2.4 [−23.3-28.1] 16.8 [−8.6-42.3] −36.2 [−61.2-(-11.2)] 4.4 [−21.8-30.5] ref	.855 .195 .005 .744
pH	73.2 [40.2-106.2]	<.001	29.6 [−33.3-92.4]	.356
BE	1.4 [0.8-2.1]	<.001	1.103 [−0.08-2.29]	.068
DO_2_	0.012 [−0.018-0.041]	.439	0.011 [−0.017-0.038]	.443

ref: reference

## Discussion

Cardiogenic shock is a life-threatening emergency with a high mortality rate despite numerous efforts in various diagnostic measures and employed treatments.^12^ Mechanical support such as VA-ECLS is increasingly used in cardiogenic shock to improve hemodynamic states quickly as it can be initiated rapidly.^13^ The survival rate of VA-ECLS for cardiac patients, as reported by the ELSO registry in January 2020, is 43%,^14^ and this is similar to our 30-day survival rate (44.2%). VA-ECLS, as a temporary mechanical circulatory support, is claimed to be a very effective therapy for providing gas exchange in acute cardiorespiratory failure.^15^

Current analysis indicated that ECLS was an effective treatment, yet survivors had more benefit on the effect of DO_2_ regarding hypoxic issues by the reduction of serial lactate levels. Results of this study identified that patients with ischemic cardiogenic shock cleared 36.2% less lactate compared to VA-ECLS patients with other indications. The pathophysiology of cardiogenic shock involves a downward spiral of ischemia, causing myocardial dysfunction, which, in turn, worsens ischemia,^16^ and both myocardial dysfunction and ischemia can cause elevated lactate level.^17^ However, the increased lactate level is more likely associated with increased tissue lactate production instead of decreased clearance.^18^ According to a previous study, cardiogenic shock patients might lead to a better survival rate due to early revascularization, especially using mechanical circulatory support devices to improve hemodynamic stability such as Impella, TandemHeart, and VA-ECLS.^19^

Lactate and lactate clearance are viable parameters reflective of ECLS outcome.^20^ It was demonstrated that high lactate levels are related to unfavorable prognoses, while rapid lactate clearance suggests adequate tissue reperfusion, increasing the likelihood of survival.^5,21^ In current patient cohort, blood lactate levels were significantly higher in non-survivors than survivors at each time of measurement, indicating that non-survivors suffered from more profound tissue hypoxia and thereby accumulated oxygen debt. Higher lactate levels suggest an inadequate lactate clearance and concomitantly ongoing tissue hypoxia. One can hypothesize that the maldistribution of blood flow together with the discrepancy between serum lactate and muscle lactate levels during shock have partially contributed to these results.^22^

Furthermore, higher pre-ECLS lactate values were associated with an increased risk of 30-day mortality. Thus, they may indicate the presence of a more severe hypoperfusion state and greater severity of pre-ECLS conditions contributing to unfavorable clinical outcome.^23^ Similar findings are described by Yang et al where lactate level above 5 mmol/L for a duration of 3.3 h correlates significantly with mortality.^24^ In contrast, earlier studies reported no association between blood lactate levels before ECLS therapy and 30-day mortality in adults with refractory cardiogenic shock.^23^

Previous studies identified that lactate dynamics in the early phase following ECLS initiation is predictive of mortality.^21,23^ Serial lactate measurements over time or lactate clearances have been reported clinically more reliable than absolute lactate values as a proxy for the magnitude and duration of global tissue hypoxia and risk stratification in different pathologic conditions ranging from sepsis to trauma.^25^ Conversely, the current study did not find evidence that lactate clearance is associated with VA-ECLS outcome. Similar results were found in a previous study in children with refractory cardiogenic shock treated with ECLS.^26^ Though ECLS established partial washout of accumulated lactate in non-surviving patients, relatively high lactate concentrations remain present compared to surviving patients. Despite the lack of a statistically significant difference, a greater decrease in lactate was noted in survivors at most time points. A possible explanation can be found in the fact that non-survivors were already in severe hyperlactatemia with tissue hypoxia before ECLS initiation, and therefore, cannot be sustained over time.^26^ This might also explain why early ECLS initiation affects lactate clearance. Yet, this study showed that lactate levels decreased on average by 1.1% per 100 ml/min/m^2^ of oxygen delivered. This finding was not statistically significant, and therefore additional research is warranted to confirm our results.

The current study revealed that blood pH in the early phase could predict the 30-day mortality in refractory cardiogenic shock patients treated by VA-ECLS support. Lower blood pH levels at 26 h after the initiation were associated with a higher risk of 30-day mortality. Previous studies reported an elevated pH (pH >7.2) before ECLS initiation is associated with an increased chance of survival.^27^ Although pH for both groups was higher than 7.2 at all time points in the current study, significantly higher pH levels were observed among survivors at each time of measurements except for the time point prior to treatment initiation. The severity of the underlying disease is assumed to contribute to lower pH levels, as well as poor oxygen delivery and organ perfusion.^28^

Physiologically, lactate clearance directly depends upon liver and kidney function. Roth et al demonstrated that elevated alkaline phosphatase and total bilirubin values are sensitive predictors of short- and long-term outcomes in patients undergoing extracorporeal membrane oxygenation following cardiovascular surgery.^29^ Also, elevated liver enzyme concentrations were only compensated for after five days of ECLS, and enzyme levels were not associated with poor outcomes.^29^ However, this study failed to show that these aforementioned liver enzymes were reliable markers for 30-day mortality. While not statistically significant, a progressive worsening in renal function in both survivors and non-survivors was observed. Acute kidney injury (AKI) is common in patients on ECLS, with an incidence as high as 70% to 85% and associated with increased mortality rates up to 80%.^30^ The pathophysiology of associated AKI is mainly related to a reduction in renal oxygen delivery and/or to inflammatory damage. Regardless of improvement of general tissue perfusion in cardiogenic shock patients, the steady blood flow during VA-ECLS may be inadequate to maintain tissue perfusion and oxygen delivery in peripheral organs such as the kidney.^31^ Possibly due to the heterogeneity of the study population and the short observational time, no significant improvements in liver or kidney functions were observed.

There are several limitations related to this study that should be acknowledged. First, inherent to the study design's retrospective single-center nature, a limited number of patients were included and data quality might be suboptimal. Therefore, future studies should aim to include patient records from multiple medical centers and prospective data collection. Secondly, the heterogeneous patient population with different ECLS indications might have affected the results. Lastly, cardiogenic shock is a complex life-threatening emergency with considerable variation in etiology, severity, and occurrence of concomitant complications. Hence, identifying the role and contribution of prognostic factors, specifically modifiable risk factors related to clinical outcome, should be prioritized in future studies to achieve optimal deployment of ECLS in the future ultimately.

## Conclusions

ECLS showed to be an effective treatment in reducing blood lactate levels in patients suffering from refractory cardiogenic shock in which the outcome is influenced by the initial lactate level and pH in the early phase of the intervention.

## Supplemental Material

sj-docx-1-cra-10.1177_11795484221113988 - Supplemental material for Efficacy of Veno-Arterial Extracorporeal Life Support in Adult Patients with Refractory Cardiogenic Shock Click here for additional data file. Supplemental material, sj-docx-1-cra-10.1177_11795484221113988 for Efficacy of Veno-Arterial Extracorporeal Life Support in Adult Patients with Refractory Cardiogenic Shock by ER Kurniawati, SMJ van Kuijk, NPA Vranken, JG Maessen and PW Weerwind in Clinical Medicine Insights: Circulatory, Respiratory and Pulmonary Medicine
