# Supplementary material for: Efficacy of Veno-Arterial Extracorporeal Life Support in Adult Patients with Refractory Cardiogenic Shock
Source: Clin Med Insights Circ Respir Pulm Med. 2022 Jul 21;16:11795484221113988. doi: 10.1177/11795484221113988 (PMC9309772; doi:10.1177/11795484221113988)
Supplement: sj-docx-1-cra-10.1177_11795484221113988 - Supplemental material for Efficacy of Veno-Arterial Extracorporeal Life Support in Adult Patients with Refractory Cardiogenic Shock [file sj-docx-1-cra-10.1177_11795484221113988.docx]

| Figure 1.  Figure 2.  Figure 1.  Figure 2.  Figure 3.  Figure 4.  Figure 5.  Figure 6.  Figure 7.  Figure 8.  Figure 9.  Figure 10.  Figure 11.  Figure 12.  Figure 13.  Figure 14. | **Appendix 1**  Blood pH during the first 26 hours of VA-ECLS  Base excess during the first 26 hours of VA-ECLS  Lactate level during the first 26 hours of VA-ECLS  AST during the first 26 hours of VA-ECLS  ALT during the first 26 hours of VA-ECLS  LDH during the first 26 hours of VA-ECLS  Bilirubin level during the first 26 hours of VA-ECLS  Creatinine level during the first 26 hours of VA-ECLS  Urea during the first 26 hours of VA-ECLS  CK during the first 26 hours of VA-ECLS  eGFR during the first 26 hours of VA-ECLS  Blood flow during the first 26 hours of VA-ECLS  Indexed oxygen delivery (DO_2_) during the first 26 hours of VA-ECLS  Oxygen delivery (DO_2_) during the first 26 hours of VA-ECLS |
| --- | --- |
| pH | Observation time (hours)  * p<0.05  Figure 1. Blood pH during the first 26 hours of VA-ECLS |
| Base excess (mmol/L) | **Base excess**  Observation time (hours)  * p<0.05  Figure 2. Base excess during the first 26 hours of VA-ECLS |
| Lactate concentration (mmol/L) | Observation time (hours)  * p<0.05  Figure 3. Lactate level during the first 26 hours of VA-ECLS |
| AST (U/L) | Observation time (hours)  * p<0.05  Figure 4. AST during the first 26 hours of VA-ECLS |
| ALT (U/L) | Observation time (hours)  * p<0.05  Figure 5. ALT during the first 26 hours of VA-ECLS |
| LDH (U/L) | Observation time (hours)  * p<0.05  Figure 6. LDH during the first 26 hours of VA-ECLS |
| Bilirubin ((μmol/L) | Observation time (hours)  * p<0.05  Figure 7. Bilirubin level during the first 26 hours of VA-ECLS |
| Creatinine ((μmol/L) | Observation time (hours)  * p<0.05  Figure 8. Creatinine level during the first 26 hours of VA-ECLS |
| Urea (mmol/L) | Observation time (hours)  * p<0.05  Figure 9. Urea during the first 26 hours of VA-ECLS |
| CK (U/L) | Observation time (hours)  * p<0.05  Figure 10. CK during the first 26 hours of VA-ECLS |
| eGFR (mL/min/1.73m^2^) | Observation time (hours)  * p<0.05  Figure 11. eGFR during the first 26 hours of VA-ECLS |
| Blood flow (L/min) | Observation time (hours)  * p<0.05  Figure 12. Blood flow during the first 26 hours of VA-ECLS |
|  | Indexed DO_2_ (mL/min/m^2^)  Observation time (hours)  * p<0.05  Figure 13. Indexed oxygen delivery (DO_2_) during the first 26 hours of VA-ECLS |
| Oxygen delivery (mL/min) | Observation time (hours)  * p<0.05  Figure 14. Oxygen delivery (DO_2_) during the first 26 hours of VA-ECLS |
